# Increased dietary protein in the second trimester of gestation increases live weight gain and carcass composition in weaner calves to 6 months of age

**DOI:** 10.1017/S1751731116002330

**Published:** 2016-11-08

**Authors:** G. G. Miguel-Pacheco, L. D. Curtain, C. Rutland, L. Knott, S. T. Norman, N. J. Phillips, V. E. A. Perry

**Affiliations:** 1 School of Veterinary Medicine and Science, University of Nottingham, Sutton Bonington LE12 5RD, UK; 2 School of Animal Studies, University of Queensland, Gatton, QLD 4345, Australia; 3 School of Veterinary Science, University of Queensland, Gatton, QLD 4345, Australia; 4 School of Animal and Veterinary Sciences, Charles Sturt University, Wagga Wagga, NSW 2650, Australia; 5 Robinson Research Institute, School of Medicine, University of Adelaide, Frome Road, SA 5001, Australia

**Keywords:** foetal programming, bovine, calf growth, gestation diet

## Abstract

Genetically similar nulliparous Polled Hereford heifers from a closed pedigree herd were used to evaluate the effects of dietary protein during the first and second trimester of gestation upon foetal, placental and postnatal growth. Heifers were randomly allocated into two groups at 35 days after artificial insemination (35 days post conception (dpc)) to a single bull and fed high (15.7% CP) or low (5.9% CP) protein in the first trimester (T1). At 90 dpc, half of each nutritional treatment group changed to a high- or low-protein diet for the second trimester until 180 dpc (T2). High protein intake in the second trimester increased birth weight in females (*P*=0.05), but there was no effect of treatment upon birth weight when taken over both sexes. Biparietal diameter was significantly increased by high protein in the second trimester with the effect being greater in the female (*P*=0.02), but also significant overall (*P*=0.05). Placental weight was positively correlated with birth weight, fibroblast volume and relative blood vessel volume (*P*<0.05). Placental fibroblast density was increased and trophoblast volume decreased in the high-protein first trimester treatment group (*P*<0.05). There was a trend for placental weight to be increased by high protein in the second trimester (*P*=0.06). Calves from heifers fed the high-protein treatment in the second trimester weighed significantly more on all occasions preweaning (at 1 month (*P*=0.0004), 2 months (*P*=0.006), 3 months (*P*=0.002), 4 months (*P*=0.01), 5 months (*P*=0.03), 6 months (*P*=0.001)), and grew at a faster rate over the 6-month period. By 6 months of age, the calves from heifers fed high nutrition in the second trimester weighed 33 kg heavier than those fed the low diet in the second trimester. These results suggest that dietary protein in early pregnancy alters the development of the bovine placenta and calf growth to weaning.

## Implications

Protein supplementation during pregnancy in nulliparous heifers may significantly increase growth rate and muscle development in the progeny. This may have significant financial implications to the cattle producer. This effect may in part be executed via the observed adaptations in the developing placenta.

## Introduction

It is well established from epidemiological studies in human populations and experimental studies in a range of animal models that varying maternal nutrition during critical periods of foetal development can alter or ‘program’ body mass and body composition in later life (Symonds *et al*., 2004; Micke *et al*., 2010a; Micke *et al*., 2011a; Micke *et al*., 2011b). Range cattle managed under extensive conditions experience such variations in maternal nutrition sufficient to affect foetal programming and thereby the postnatal growth and carcass characteristics of their progeny (Cafe *et al*., 2006; Greenwood *et al*., 2006; Martin *et al*., 2007; Micke *et al*., 2010a; Summers *et al*., 2015). Maternal nutrient supply to the foetus regulates the foetal IGF axis (Oliver *et al*., 1996; Sullivan *et al*., 2009a) and can programme the postnatal IGF axis (Micke *et al*., 2010a; Symonds *et al*., 2012). Calf plasma IGF-1 at birth is positively associated with birth weight (Breier *et al*., 1988; Micke *et al*., 2010a), as is postnatal IGF concentration with average daily gain (ADG) and linear growth (Lund-Larsen *et al*., 1977; Micke *et al*., 2010a; Micke *et al*., 2010b). The reported effects of foetal programming upon ADG and carcass development in cattle progeny is however inconsistent (Greenwood *et al.*, 2005; Micke *et al*., 2011b; Summers *et al*., 2015). The disparity recorded between these studies may be influenced by the genetic heterogeneity, and age of the dams observed as well as timing of intervention: the Greenwood *et al.* (2005) study used both pluriparous and nulliparous dams, whereas the Micke *et al*. (2011b) and Summers *et al*. (2015) only nulliparous, with each study using different relatively diverse genotypes and intervention periods.

In this study, nulliparous Polled Hereford heifers from a closed stud herd mated at 15 months of age were used, ensuring a reduction in genetic variation within the dams and enabling focus upon the adolescent yearling heifer which has been shown to be more susceptible to foetal growth restriction following gestational nutritional perturbation (Copping *et al*., 2014; Hernandez-Medrano *et al*., 2015). Based on our former studies (Perry *et al*., 1999; Sullivan *et al*., 2009b; Micke *et al*., 2010a; Micke *et al*., 2015), we hypothesise that low protein in the first trimester will enhance placental development and in the second trimester will result in reduced growth and carcass muscling in the offspring.

## Materials and methods

### Project animals management and treatments

All procedures were performed with the approval of the University of Queensland Animal Ethics Committee, approval number SVS/748/08. Genetically similar Polled Hereford heifers (*n*=80), ~15 months old from the same closed pedigree herd, were selected for inclusion in this trial. Preceding artificial insemination, the heifers were weighed and their reproductive tract palpated for normality. Oestrus was synchronised by insertion of intravaginal progesterone implants (‘EAZI-BREED CIDR-B’; Genetics Australia, Bacchus Marsh, VIC, Australia) for 11 days, with an injection of 12.5 mg of dinoprost tromethamine (Lutalyse; Upjohn Pty Limited, Rydalmere, NSW, Australia) and 500 IU of equine chorionic gonadotrophin (ECG) on the day of CIDR removal. Approximately 48 h after the CIDR implants were removed, the heifers were all artificially inseminated on the same day without detection of oestrus. The sire used was a Polled Hereford bull with an estimated breeding value for birth weight of +2.1 kg at 86% reliability.

The study design was a 2×2 factorial design. The heifers were stratified by body weight and randomly allocated to two equal first trimester (T1) dietary treatment groups, high (15.7% CP) and low (5.9% CP) protein at 14 days post conception (dpc). Pregnancy diagnosis was completed by manual palpation at 35 days following insemination (taken as 35 dpc). As T1 treatment did not start at conception, the periconception period is, therefore, not addressed. After pregnancy diagnosis, all non-pregnant animals were removed from trial (*n*=21). Following an outbreak of Bovine Ephemeral Fever during trimester 1, 17 heifers were recorded to have aborted and were excluded from the study. The remaining numbers of calves in each treatment period were: T1 high protein, *n*=10 consisting of six male and four female calves; and T1 Low protein, *n*=11 consisting of seven male and four female. At 90 dpc half of each nutritional group was changed to a high (15.6% CP) or low (6.1% CP) protein treatment until 180 dpc (T2). Numbers of animals in T2 high protein: *n*=13 consisting of 10 male and three female; and in T2 low protein: *n*=8 consisting of three male and five female. This factorial design gave rise to four treatment groups: high/high (HH; four males and two female), high/low (HL; two males and two females), low/high (LH; six males and one female), low/low (LL; one male and three females). After 180 dpc heifers were run together on the same native pasture during the final trimester until term. The diet for this final trimester consisted of native pasture, containing grasses and *medicago polymorpha* with a CP value ranging between 10% and 13% over the trimester. Both dietary groups of heifers received a grain ration and either *ad libitum* pasture hay (low) or pasture grazing (high). Details on dry matter intake (DMI) calculations are given in (Table 1).

**Table 1:** Nutrient content of dietary rations fed to dams during each trimester of gestation (T1=36 to 90 dpc, T2=90 to 180 dpc or T3=181 to term) by treatment (high or low)

	T1		T2		T3
	(36 to 90)		(90 to 180)		181 to term
Feed sources	High	Low	High	Low	All cows
Hay (kg)	–	3.74	–	4.00	–
Pasture (kg)	5.72	–	6.00	–	7.20
Sorghum (kg)	–	2.61	–	2.61	–
Copra meal (kg)	1.80	–	1.80	–	–
Calcium (g/kg)	1.10	2.60	1.10	2.60	1.10
Phosphorus (g/kg)	9.20	5.20	9.20	5.20	9.20
DMI total^1^	7.52	6.35	7.80	6.61	7.20
Energy intake (MJ)	70.50	57.50	72.20	64.00	63.40
Energy (%NRC^2^)	100.00	90.00	103.00	92.00	101.00
CP (kg)	1.18	0.38	1.22	0.40	0.99
CP (%)	15.70	5.98	15.64	6.05	13.70
CP (%NRC)	174.00	66.00	179.00	70.00	125.00

Data are presented on an as-fed dry matter (DM) basis per heifer per day.

1 Estimates of DMI from pasture and hay were calculated based upon the National Research Council (NRC) energy requirements for replacement *Bos taurus* pregnant heifers with a mature weight of 500 kg and a calf birth weight of 32 kg. As the sorghum and copra meal energy content were known (12 and 11.2 MJ/kg DM, respectively) and the heifers on the low-protein diet averaged 340 kg with a rate of gain of 0.8 kg during the first trimester, the DMI of pasture and hay was calculated based upon the energy requirement sufficient to sustain this rate of gain. Similar DMI estimations were completed in the second and third trimesters based on the rate of gain in each treatment group.

2 NRC comparison to ration to NRC (1996) recommended nutrient requirements for pregnant yearling *Bos taurus* replacement heifers with calf weight of 32 kg.

Before calving, heifers were individually placed in a small yard. Measurements were taken of the newborn calf before suckling (birth weight (BIRW) and biparietal diameter (BPD)), and the foetal portion of the placenta was collected immediately upon expulsion. Placental measures included wet weight, cotyledon number and wet weight of cotyledons (CWW) post separation from adjoining membranes. The dry cotyledon weight (CDW) measure was obtained by drying cotyledons overnight at 100°C and then weighing at 1 h intervals, until the same weight was recorded on three consecutive weightings.

After calving, all the heifers were grazed together with their calves on native pasture. Weights from the heifers and calves were recorded monthly. At 6 months of age, ultrasound (model Aloka-500^®^; Aloka Inc., Tokyo, Japan with 3 MHz linear probe) was used to assess fat depth at the P8 (rump) and 12th rib (FT12) sites (Hopkins, 1989). Anal Fat Fold (AFFT), the thickness of skin and subcutaneous fat situated between the point of the ischium and the base of the tail, was assessed using techniques described by Johnson (1994) by a single experienced technician using calibrated calipers. Heifers were fasted for 6 h and weighed to obtain their empty live weight (ELW). ELW and AFFT were used to calculate the percentage carcass components (muscle, fat and bone) following the published regression equations in *Bos taurus* Hereford cattle by Johnson (1994).

### Quantitative analysis of placental tissue

The placenta was immediately collected after expulsion (Stage 3 of labour varying between 0.5 and 6 h) at term and weighed whole before excision of all cotyledons from the surrounding membranes (Perry *et al*., 1999). Those not completely expelled by 6 h post calving were considered retained foetal membranes and not used. One small (average 4.4 g), one medium (average 24.2 g) and one large cotyledon (average 55 g) were removed from the gravid horn, fixed in neutral buffered formalin (10%) for 48 h, processed and embedded in paraffin wax. Two-micron sections were cut from each cotyledon and stained with haematoxylin–eosin (H&E) with a further section stained with Masson’s Trichrome.

Quantitative analysis was used to calculate volume densities and relative total component volumes of major cellular components, surface density and barrier thickness of trophectoderm. An L-36 Merz grid was used in conjunction with a video image analysis system as detailed by Perry *et al*. (1999) for morphometric analysis, at a final magnification of 250× (Nikon 80i microscope with Nikon DS camera system software; Nikon, Tokyo, Japan). The points falling on each structure or component were manually assigned using morphological features appropriate to H&E or Masson’s Trichrome as appropriate, blood vessels were not differentiated between arteries, veins or capillaries. Ten systematic random fields of each cotyledon, of each size, were analysed (three cotyledons per cow), thus 30 fields were analysed per animal to calculate structural quantities (Weibel *et al*., 1966). The cellular components that were measured and counted by point and intersection counting were: trophectoderm, connective tissue matrix, connective tissue fibroblasts and connective tissue blood vessels.

Equations detailed by Weibel *et al*. (1966), were used to calculate relative volume densities of each component, surface density and mean barrier thickness of the foetal trophectoderm. The relative volume densities (calculated at 1 g of placenta occupying 1 cm^3^) in conjunction with the CWW (collected as detailed above) of each placenta were used to calculate relative total component volumes of cellular components. The surface area of the foetal trophectoderm was also calculated from the number of times the lines on the Merz grid intersected the surface of the trophectoderm. Volume density=number of points falling on structure/total number of test points (*V*_*d*_=*P*_*a*_/*P*_*T*_). Relative volume of each component=volume density×placental weight in grams (*V*_*c*_=*V*_*d*_×weight (g)). Surface density=2×number of intercepts/total length of lines (*S*_*v*_=2×*L*_*a*_/*L*_*T*_). Barrier thickness=trophoblast volume density/surface density of the trophoblast (*B*_*T*_=*D*_*d*_/*S*_*v*_) (Weibel *et al*., 1966; Roberts *et al*., 2001). Every photomicrograph was high quality and could be counted, resulting in 1080 point counts per animal.

### Statistical analyses

The study used a 2×2 factorial design, with factors being nutrition in the first trimester (T1) and the second trimester (T2). Statistical analyses were performed using StataCorp. 2015 (*Stata Statistical Software: Release 14*; StataCorp LP, College Station, TX, USA), and significance was set at *P*<0.05 for all the results. Normality and equality of variance was checked before any analysis and transformation of the data was applied if it was necessary.

Birth calf weights were analysed using ANOVA with T1×T2, with sex used as covariate. Birth weight and the monthly calf weights were all subjected to ANOVA with repeated measures, and sex and calf age were added as covariates. For each calf the ADG (kg/day) between birth and the final weighing was computed. This calculation involved just the first and last set of weights. The linear growth rate (g/day), which involved fitting a straight line to the weight data and estimating the slope, was also calculated yielding the linear growth rate. Both calculations gave very similar results with live weights and growth rates showing the same response to the treatments.

Muscle, fat and bone in the live animal were considered as three separate dependent variables, against the fixed effects of nutrition in T1×T2 and sex. The effect of group was also analysed. ANOVA was used to examine treatment effects upon placental tissues adding calf sex as covariate. Correlation coefficients between treatment effects and other parameters were also calculated using Pearson’s correlation coefficient and were also considered statistically significant if *P*<0.05.

## Results

### Placental gross morphometry

There was a trend for placental weight to be increased in the high-protein second trimester treatment group (*P*=0.06), but there was no significant effect upon CWW and CDW. In addition, no significant differences were observed between treatments in relation to cotyledon number (Table 2).

**Table 2:** Placental gross morphometry and calf measurements at birth by treatment group

First trimester	High protein				Low protein			
Second trimester	High		Low		High		Low	
	Mean	SEM	Mean	SEM	Mean	SEM	Mean	SEM
Placental gross morphometry								
Number of cotyledons	78.0	±6.9	98.5	±18.5	78.5	±7.3	70.3	±8.2
Cotyledon wet weight (g)	1493.3	±82.7	1308.5	±91.5	1544.3	±165.7	1335.0	±163.5
Cotyledon dry weight (g)	124.3	±17.1	88.8	±22.7	128.4	±20.5	130.6	±12.4
Placental wet weight (g)^1^	4923.8	±356.5	3935.0	±585.0	4247.0	±367.9	3405.5	±561.2
Calves measurements								
Birth weight	36.8	±0.9	31.0	±1.8	36.7	±2.3	36.0	±1.9
BPD	10.8^b^	±0.1	10.5^a^	±0.2	10.7^b^	±0.2	10.3^a^	±0.2

BPD=biparietal diameter. ^a,b^Different alphabetic superscripts denote mean values which are significantly different from each other (*P*<0.05).

1 The observed increase in placental weight by second trimester high-protein treatment was below this level at *P*=0.06.

### Placental cellular components

Fibroblast density was increased by the high-protein treatment in the first trimester (*P*=0.01; Table 3), whereas low protein intake in the first trimester increased trophectoderm volume (*P*<0.05; Table 3). There were, however, no significant effects of treatment upon other density measures of cellular components, matrix density and relative blood vessel volume density (BVVD). There were no statistically significant interactions between treatments for any of the cellular components measured (Table 3).

**Table 3:** Cellular composition of bovine placenta by treatment group

First trimester	High protein				Low protein			
Second trimester	High		Low		High		Low	
	Mean	SEM	Mean	SEM	Mean	SEM	Mean	SEM
Cellular composition of bovine placenta								
Surface density of trophectoderm (cm^-1^)	202.8	±7.5	205.1	±17.4	202.5	±15.06	210.4	±25.5
Barrier thickness of trophectoderm (cm)	0.233	±0.015	0.247	±0.014	0.237	±0.015	0.262	±0.042
Volume densities (cm^0^)								
Trophectoderm	0.485	±0.026	0.511	±0.029	0.473	±0.027	0.526	±0.023
Matrix	0.398	±0.021	0.381	±0.012	0.358	±0.042	0.381	±0.031
Fibroblast	0.038^a^	±0.004	0.035^a^	±0.002	0.031^b^	±0.002	0.025^b^	±0.004
Blood vessel volume density	0.092	±0.015	0.087	±0.023	0.091	±0.009	0.066	±0.016
Relative volume of each component (cm^3^)								
Trophectoderm volume	653.9^a^	±73.4	636.6^a^	±7.4	723.5^b^	±68.6	709.02^b^	±112.9
Matrix volume	597.7	±52.6	484.2	±43.6	574.0	±106.4	497.3	±41.57
Fibroblast volume	57.04	±7.69	48.41	±3.38	47.61	±6.37	32.05	±5.64
Blood vessel volume	141.3	±26.9	141.3	±37.9	146.6	±31.3	95.3	±32.2

Values are unadjusted mean and ±SEM of the cellular composition of bovine placenta by treatment group. Volume densities are a proportion, dimensionless numbers as they are a ratio of two numbers=cm^0^; Surface density measures surface area per volume or weight of tissue (g of cotyledon) (cm^2^/cm^3^)=cm^−1^; Barrier thickness of trophectoderm – linear measurement=cm; relative volume of each component assumes 1 g of placenta occupies 1 cm^3^=cm^3^. ^a,b^Different alphabetic superscripts denote mean values that are significantly different (*P*<0.05) from each other.

### Calf measures at birth

High protein in the second trimester significantly increased BPD (*P*=0.05; Table 2), with a greater effect in female progeny (*P*=0.02). There was a similar effect of second trimester high-protein diet increasing birth weight in female progeny (*P*=0.05), however no effect of treatment on overall birth weight. There was no interaction between treatment and sex. There was increased variability in the birth weight of male calves.

### Relationships between birth weight and placental parameters

BIRW was positively correlated with placental weight, CWW, and cotyledon number, with a tendency for a positively correlation with CDW. The strongest correlation being with CWW (*r*=0.65, *P*=0.003; Table 4). Relative matrix volume (*P*=0.008) and relative blood vessel volume (BVV) (*P*=0.03) were the only cellular parameters correlated with BIRW (Table 4). Cotyledon number was highly positively correlated with BVVD (*r*=0.569, *P*=0.014), matrix volume (*r*=0.497, *P*=0.036), fibroblast volume (*r*=0.594, *P*=0.009), BVV (*r*=0.575, *P*=0.012) and CWW (*r*=0.531, *P*=0.023); and negatively correlated with surface density (*r*=−0.605, *P*=0.008) and trophectoderm volume density (*r*=−0.493, *P=*0.037). BPD had strong positive correlations with BVVD (*r*=0.674, *P=*0.002), matrix volume (*r*=0.685, *P=*0.002), BVV (*r*=0.769, *P*=0.0002), BIRW (*r*=0.678, *P*=0.001), CWW (*r*=0.783, *P*=0.0001), CDW (*r*=0.511, *P*=0.03) and placental weight (*r*=0.679, *P*=0.001). Placental weight was significantly positively correlated with matrix volume (*r*=0.653, *P*=0.003), fibroblast volume (*r*=0.498, *P*=0.035), BVV (*r*=0.522, *P*=0.03) and BVVD (*r*=0.478, *P*=0.04).

**Table 4:** Correlation coefficients (*r*) between birth weight and placental parameters

Variables	*r*	*P*
Placental parameters		
Placental weight	0.52	0.03
CWW	0.65	0.003
CDW	0.43	0.07
Cotyledon number	0.46	0.05
Cellular placental parameters		
Trophectoderm relative volume	0.392	Ns
Matrix relative volume	0.601	0.008
Fibroblast relative volume	0.331	Ns
Blood vessel relative volume	0.518	0.03
Trophectoderm volume density	−0.242	Ns
Matrix volume density	0.249	Ns
Fibroblast volume density	−0.022	Ns
Blood vessel volume density	0.439	0.06
Surface density of the trophectoderm	−0.396	Ns
Barrier thickness of trophectoderm	0.114	Ns

CWW=cotyledon wet weight; CDW=cotyledon dry weight; Ns=not significant.

### Preweaning growth of calves

**Figure 1:**
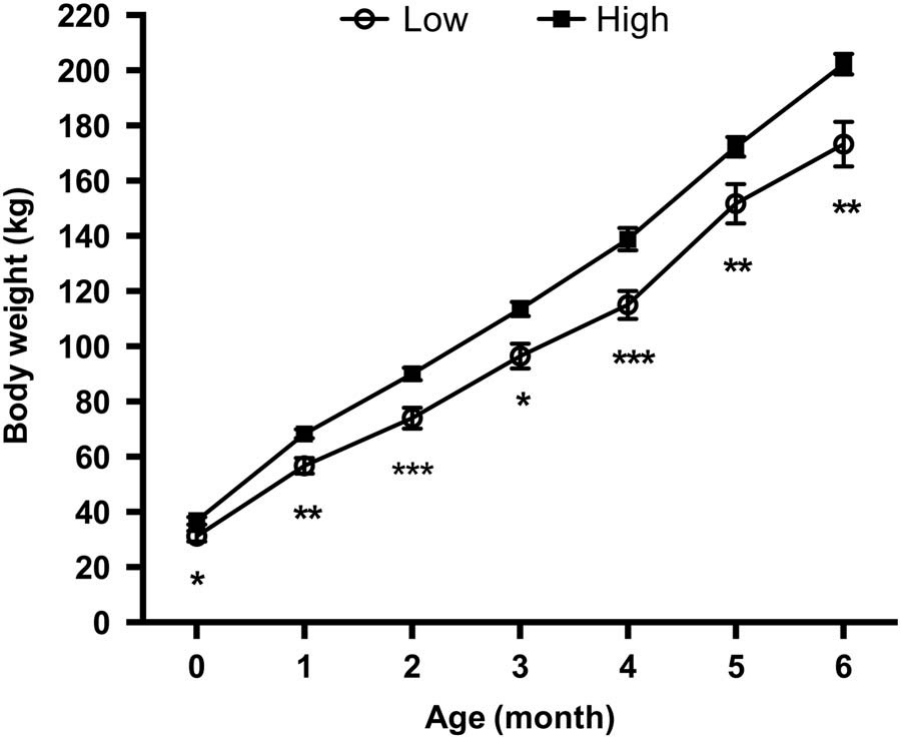
Mean and ±SEM of calf body weight from birth (0 months) until 6 months of age by treatment during the second trimester. Levels of significance indicated by *P<0.05, **P<0.01 and ***P<0.001, respectively.

Calf live weights and growth rates showed a similar response to the treatments, whereby there was no effect of nutrition in trimester one on ADG, and there was no sex effect observed on ADG and no interaction between protein levels fed in trimesters one and two (*P*>0.05). There was a significant effect of nutrition in trimester 2 on ADG (*P*=0.01) (Figure 1). Calves from heifers fed the high-protein diet in the second trimester weighed significantly more on all occasions (at 1 month (*P*=0.004), 2 months (*P*=0.006), 3 months (*P*=0.002), 4 months (*P*=0.01), 5 months (*P*=0.03), 6 months (*P*=0.01), and grew at a faster rate over the 6-month period (Figure 1). By 6 months of age, the calves from the heifers fed the high-protein diet in the second trimester weighed 33 kg heavier than those fed the low diet in the second trimester (16% heavier).

In the live carcass measures, there was a sex effect on the amount of muscle (*P*=0.0001) and fat (*P*=0.0007), but not bone (*P*=0.91); males had increased muscle (59.67±0.26) and less fat (23.15±0.38) compared with females (muscle: 57.04±0.51, and fat: 26.71±0.76). For all three variables, there was a significant effect of nutrition in trimester 1 and a significant interaction between nutrition fed in the two trimesters. Decreased protein during trimester 1 led to increased muscle (*P*=0.002) and decreased fat (*P*=0.002). Animals in the HL group had significantly lower muscle and bone percentages and significantly higher fat percentages than animals in the LL group. No other group was different from another in either muscle bone or fat (Table 5).

**Table 5:** Anal fat fold (AFFT) measurement and body composition of calves at 6 months of age according to treatment groupEquations used to calculate the body composition were taken from Johnson (1994). Different alphabetic superscripts denote group mean values that are statistically significantly different from each other, a, b and c for *P*<0.05

First trimester	High protein						Low protein					
Second trimester	High			Low			High			Low		
	Mean	SEM	CI	Mean	SEM	CI	Mean	SEM	CI	Mean	SEM	CI
Fat measurements												
AFFT (mm)	22.30	±1.52	18.42 to 26.24	35.75	±1.11	32.22 to 39.28	26.28	±2.16	21.01 to 31.56	20.00	±1.41	15.49 to 24.50
Body composition												
Muscle % (1)	58.71^ab^	±0.76	56.75 to 60.68	57.00^a^	±0.88	54.19 to 59.81	59.43^ab^	±0.55	58.08 to 60.77	58.92^b^	±0.62	56.93 to 60.91
Fat % (2)	24.42^ab^	±1.06	21.69 to 27.15	26.90^a^	±1.25	22.91 to 30.89	23.50^ab^	±0.77	21.62 to 25.38	24.00^b^	±0.85	21.29 to 26.70
Bone % (3)	14.03^ab^	±0.13	13.71 to 14.36	13.60^a^	±0.16	13.09 to 14.10	14.08^ab^	±0.12	13.78 to 14.39	14.55^b^	±0.87	14.27 to 14.82

Equations used to calculate the body composition were taken from Johnson (1994). (1)=72.92 (if males)/69.97(if females)−0.090×AFFT−0.027×ELW; (2)=4.37(if male)/8.32 (if female)+0.132×AFFT+0.038×ELW; (3)=18.81−0.029×AFFT−0.010×ELW. ^a,b,c^Different alphabetic superscripts denote mean values that are statistically significantly different from each other for *P*<0.05.

## Discussion

This study provides clear evidence that nutrition during the first two trimesters of pregnancy alters the growth and body phenotype of the calf. Importantly, decreasing genetic variability in the heifer dams enabled this clear effect of gestational protein manipulation upon progeny growth to be revealed. The analysis of weight gain of progeny supports the hypothesis that providing heifers with a high-protein diet in the second trimester resulted in a faster growth rate and a significant increase in body weight (~33 kg) in their calves at 6 months of age when compared with calves born to heifers fed low protein in the second trimester. Further, this study also shows early gestational dietary protein affects the developing placenta and supports the hypothesis that reduced protein in the first trimester increases physiologically important components of placental development. These findings are of significant interest to the cattle industry in Australia as protein is the most deficient nutrient in the Australian rangelands (Norman, 1963; Sullivan *et al*., 2009a).

This downside to utilising these high-value pedigree animals and the farm site of this study was a reduction in the level of intervention permitted. This included prevention of dietary intervention before 35 dpc, which is known to affect foetal development (Copping *et al*., 2014; Hernandez-Medrano *et al*., 2015).

Poor maternal dietary protein in early gestation has been shown to impair bovine foetal growth (Long *et al*., 2009; Micke *et al*., 2010b; Copping *et al*., 2014), which was confirmed in this experiment. Low dietary protein intake in the second trimester decreased BPD similar to previous experiments although birth weight was not affected as comprehensively as previously reported (Cafe *et al*., 2006; Micke *et al*., 2010c), being only significant in the female. The relatively small numbers in this experiment compared with the Micke paper (Micke *et al*., 2010c) may have influenced this result as variability was greater in the male calves. Furthermore, birth weight at term may not be indicative of intrauterine growth restriction (IUGR) in earlier gestation (Long *et al*., 2009; Hernandez-Medrano *et al*., 2015) in the bovine. BPD and BIRW had a strong positive relationship with placental weight and the placental parameters of BVV, CWW and CDW, concomitant with previous studies (Perry *et al*., 1999; Sullivan *et al*., 2009b). There was a trend (*P*=0.06) for placental weight to be increased by increased dietary protein in the second trimester, whereas trophectoderm volume was increased by high-protein first trimester as previously described (Perry *et al*., 1999). The initial rapid growth of the placenta has been linked to the rapid and vigorous development of the foetal trophoblast through proliferation and branching of the foetal villi into the maternal stromal tissue (Bell *et al*., 1999). Interdigitation between the foetal trophectoderm and maternal microvilli is complete by 28 dpc in the bovine (Wooding and Beckers, 1987), which is before the initiation of our dietary treatments. Placentome weight and surface area in the bovine however, continually increase until term unlike the ovine in which placentome weight is constant or decreasing from 65 dpc (Baur, 1977; Reynolds and Redmer, 1995). The observed increase in trophectoderm volume in the low-protein heifers during the first trimester may enhance the functional potential of the placenta during this period of maximal villi development and thereby enable an increased nutrient supply to the foetus during later gestation if protein availability increases. Supporting evidence suggesting that the protein restricted bovine foetus signals to the placenta an increased requirement for nutrients is found in its ability to increase blood supply via the uterine artery (Hernandez-Medrano *et al*., 2015) and increased cotyledonary vasculature (Zhu *et al*., 2007) at 125 dpc.

The relative vasculature proportions in the foetal cotyledons were not altered in this study, although there was a positive correlation between calf birth weight and vasculature. The literature shows that vascular components of the placenta vary greatly depending upon environmental perturbations, dam age, the timing of perturbation and timing of cotyledon excision. Gestational undernutrition (Vonnahme *et al*., 2007) found decreased bovine cotyledonary vasculature in the later, but not early stages of placental development, whereas Zhu *et al*. (2007) showed nutrient restriction increased cotyledonary vascularity at day 125 with no affect at day 250. Furthermore, cows in their third or greater pregnancy did not show effects upon placental composition observed in younger heifers (Long *et al*., 2009). This may illustrate a window and age-specific effect of nutrition upon placental vascularisation.

Connective tissue fibroblast density was increased (*P*<0.05) in the placenta of heifers receiving the high-protein dietary treatment in the first trimester (HH, HL) a similar finding to that of a previous study by Roberts *et al*. (2001) in sheep, where maternal dietary restriction (70% of recommended intake) reduced the total placental surface area for exchange, and the surface density of trophoblast. However, the arithmetic mean barrier thickness for diffusion in this study was increased by this maternal food restriction (Roberts *et al*., 2001). Similarly, in the current study barrier thickness of the trophectoderm was increased by protein restriction. It has been suggested by Perry *et al*. (1999) that a restriction in protein in the first trimester in heifers may lead to a larger placenta at term due to the greater development of the microvilli, particularly if this early period of restriction is followed up by a phase of improved nutrition. Indeed the LH group did have the largest CWW (Table 2), but this was only significantly greater than CWW in the HH group (*P*=0.03). It has been suggested (Talbot *et al*., 2000; Shimada *et al*., 2001; Dunlap *et al*., 2006) that trophoblasts from ungulates (including cattle), proliferate in the absence of fibroblast growth factors (FGFs), suggesting that the role of FGFs is more limited in maintaining the trophoblast lineage in cattle in comparison with rodents. It has also been shown that fibroblasts and FGFs play a role in angiogenesis, including within the placenta (Klagsbrun and D’Amore, 1991), therefore a concomitant increase in vasculature within these placentae may be expected.

These results suggest that maternal diet restriction affects the structure and function of the placenta as previously reported (Perry *et al*., 1999; Long *et al*., 2009; Sullivan *et al*., 2009b; Sullivan *et al*., 2009c). Such perturbations to placental development may reduce foetal growth due to decreased nutrient transport via the placenta (Sullivan *et al*., 2009c) generating a decrease in birth weight (Perry *et al*., 1999; Micke *et al*., 2010c), or alternatively dichotomous placental development (as in the LH group) may be attendant to increased birth weight and associated dystocia.

Interpretation of the carcass composition results requires consideration of the stages of foetal bovine muscle development, which are predominantly controlled by IGF-2 expression that peaks between 150 and 160 dpc (Gerrard and Grant, 1994; Florini *et al*., 1996). The first wave of differentiation however occurs much earlier when primary skeletal muscle fibres (type I) differentiate from primary myotubes at 39 dpc (Robelin *et al*., 1993), the second at 90 dpc results in secondary fibres; and a third at 110 dpc gives rise to tertiary fibres (Gagniere *et al*., 1999). These critical events occur during the dietary treatments imposed in this study.

As myocytes are formed from a pool of pluripotent stem cells (Oksbjerg *et al*., 2004) extrauterine signals such as those regulated by maternal nutrient intake, may affect the number of cells committed to myoblast formation whilst also affecting the rate of myoblast proliferation and thus final myofibre number. It has previously been reported that gestational dietary regimens effectively alter placental signalling hormones (Sullivan *et al*., 2009c; Summers *et al*., 2015). These may act to signal foetal skeletal muscle IGF messenger RNA (mRNA) expression and fibre development during the first two trimesters of gestation as previously shown in foetal skeletal sheep muscle where nutritional restriction increased IGF-2 mRNA expression (Brameld *et al*., 2000) and increased IGF receptor activity (Symonds *et al*., 2012). Myofibre density is also reduced by maternal nutrient restriction (Costello *et al*., 2008). As primary fibres provide the scaffolding for secondary fibre formation, a reduction of primary fibre density resulting from decreased maternal nutrient intake during the first trimester may not be fully compensated for by the provision of increased maternal nutrient intake during the latter stages of gestation. In support of this (Micke *et al*., 2011b), found that the majority of effects of maternal protein intake upon IGF mRNA expression in adult progeny skeletal muscle occurred during the first trimester of gestation suggesting these early stages of foetal development to be the most sensitive to altered nutritional environment. This is attributed to the high rate of cellular differentiation and proliferation during the early stages of gestation compared with the high rate of cellular hypertrophy during the latter stages (Brameld *et al*., 2000; Tong *et al*., 2009). This study is a corollary of previous findings (Micke *et al*., 2011b) as decreased protein during trimester 1 (LL and LH combined) increased muscle (*P*=0.002) and decreased fat (*P*=0.002) in progeny compared with those animals whose dams received high protein during this period. Though, the predicted values for carcass components presented in this paper are representative of the data presented in Johnson (1994), it is necessary to acknowledge that these predicted measurements may be limited in their inference and accuracy when compared with ‘real’ carcass measures taken at slaughter.

The treatment effects upon carcass muscle and fat in this study reflect the reciprocal relationship between the development of muscle and adipose tissue, previously reported (Micke *et al*., 2010a) whereby the *in utero* diet alters differentiation of the mutual precursor cells of adipocytes and myocytes (Symonds *et al*., 2012). Furthermore, Micke *et al*. (2011a) also indicated a possible causal mechanism via the effect of maternal diet upon adipogenic gene expression. The exposure of the developing calf foetus to a high maternal protein intake during the first trimester may result in a relative increase in myostatin gene expression in skeletal muscle as in other species (Liu *et al*., 2011) and this may result in a decrease in muscle fibre number and an increase in the commitment of stem cells within the muscle to form preadipocytes. This effect may, however, be offset in part by an upregulation of the expression of IGF-1 receptor (IGF-1R) as discussed above which occurs in the progeny of such heifers (Micke *et al*., 2011b).

In this study the lowest muscling occurred in the HL group (*P*=0.05). As the secondary muscle fibres are the largest contributor to postnatal muscle mass of cattle and begin to form at 90 dpc (Robelin *et al*., 1993) nutrient restriction during the second trimester may have decreased formation of secondary muscle fibres during myoblast proliferation as reported in sheep (Quigley *et al*., 2005). Importantly, however, both circulating maternal and progeny IGF-1 has been shown to increase following gestational high dietary protein (Perry *et al*., 2002; Sullivan *et al*., 2009a; Micke *et al*., 2010a). This combined effect in the progeny that experienced increased protein *in utero* during the second trimester may have produced the increased growth rates observed.
